# Supercharged chimeric antigen receptor T cells in solid tumors

**DOI:** 10.1172/JCI162322

**Published:** 2022-08-15

**Authors:** Ayush Pant, Christopher M. Jackson

**Affiliations:** 1 The Bloomberg-Kimmel Institute for Immunotherapy, The Sydney Kimmel Comprehensive Cancer Center and; 2 Department of Neurosurgery, The Johns Hopkins University School of Medicine, Baltimore, Maryland, USA.

## Abstract

Chimeric antigen receptor (CAR) T cells have demonstrated success in treating select hematological malignancies, but their activity in solid tumors has been comparably modest. Challenges specific to treating solid tumors include trafficking and distribution throughout the tumor site, overcoming the immunosuppressive tumor microenvironment (TME), and identifying antigenic targets that are widely expressed and indispensable to tumor biology. In this issue of the *JCI*, Tian et al. describe the use of bicistronic CAR T cells that target multiple antigens expressed in neuroblastoma to overcome antigenic heterogeneity. Combining this approach with interventions that enhance T cell trafficking and prevent acquired dysfunction in the TME may lead to a long-awaited breakthrough in the clinical implementation of CAR T cells for the treatment of solid tumors.

## Challenges associated with chimeric antigen receptor T cell therapy in solid tumors

Chimeric antigen receptor (CAR) T cells are generated by harvesting a patient’s T cells and engineering them to express synthetic receptors composed of 3 key regulatory elements: (a) an extracellular single chain variable fragment (scFv) that confers extracellular target-recognition specificity, derived from the target-binding domain of antibodies, (b) a hinge domain and transmembrane spacer that provides flexibility and stability, and (c) an intracellular domain that triggers a signaling cascade that promotes T cell survival, proliferation, and cytotoxicity (1). First generation CAR T cells possessed scFv, which could bind to extracellular tumor associated antigens (TAAs), triggering intracellular signaling from the T cell surface glycoprotein CD3ζ chain region. Second and third generation CAR T cells linked CD3ζ domains to 1 (second generation) or 2 (third generation) costimulatory receptors consisting of CD28 and/or TNFR family members 4-1BB and OX40. While CAR T cell therapy has generated dramatic responses in hematological malignancies, such as B cell leukemias and lymphomas, it has yet to show success in clinical trials involving solid tumors (2, 3).

Impediments to CAR T cell activity in solid tumors can be attributed to several key characteristics: trafficking into the tumor, an immunosuppressive environment, heterogenous antigen expression, intracellular antigens, and immune exhaustion (4). While CAR T cells against leukemia antigens can find their targets in circulation, CAR T cells against solid tumors need to successfully penetrate the tumor and persist in a hostile environment. Expression of appropriate adhesion and chemokine receptors on T cells and their ligands on tumor-associated vasculature is a necessary step in delivering CAR T cells to their targets. Once inside the tumor, CAR T cells encounter an immunosuppressive tumor microenvironment (TME) consisting of regulatory T cells, myeloid-derived suppressive cells, cancer associated fibroblasts, and cancer cells, which collectively promote CAR T cell dysfunction. Antigen availability is also more limited in solid tumors. While CAR T cells targeting CD19 have been successful owing to plentiful extracellular expression of CD19, tumor antigens in solid tumors are often intracellular, and thus inaccessible to CAR T cells (unlike endogenous tumor-specific T cells that can recognize intracellular peptides in the context of MHC I presentation with their T cell receptors) (5). As such, CAR T cells in solid tumors are typically unable to clear the tumor completely, and immunoediting mechanisms can further thwart CAR T efficacy by removing immunogenic antigens from cancer cells or downregulating MHC I expression (6). In this issue of the JCI, Tian et al. describe a strategy to identify and engineer bicistronic CAR (BiCisCAR) T cells that recognize 2 variably expressed antigens to overcome the challenge of TAA heterogeneity in neuroblastoma (7). CAR T cells can activate a tolerogenic response similar to endogenous tumor-infiltrating lymphocytes; CAR T cells undergo activation-induced upregulation of regulatory immune checkpoints such as PD-1 and CTLA-4, which can dampen antitumor response (8). Successful CAR T therapy will require ways to prevent eventual immune exhaustion.

## Overcoming tumor antigen heterogeneity with BiCisCAR T cells

**Figure 1:**
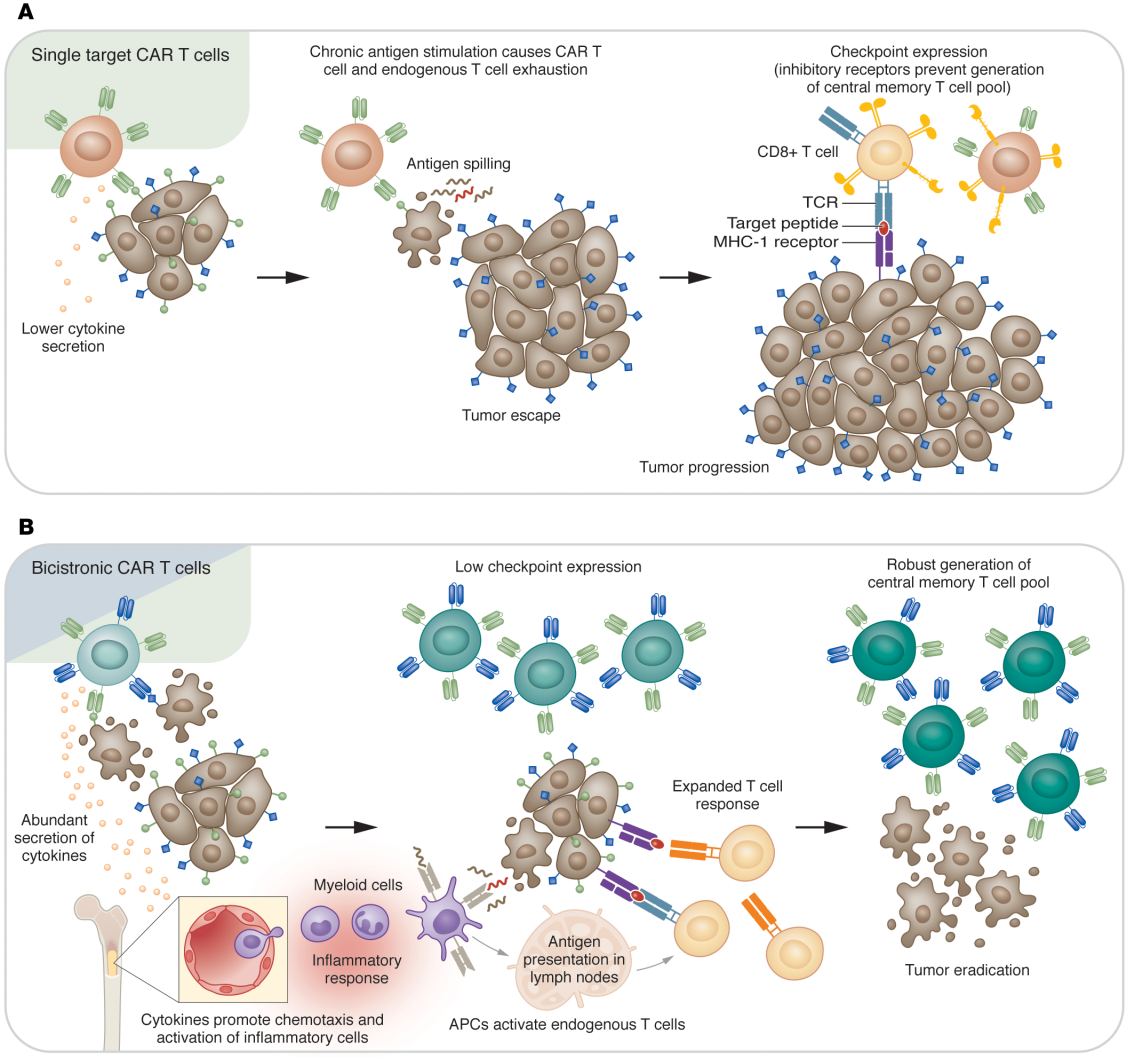
BiCisCAR T cells have enhanced efficacy against solid tumors. (**A**) Single target CAR T cells recognize and kill cancer cells expressing their target antigen. However, tumor cells that do not express or downregulate the targeted antigen are resistant to killing by CAR T cells and proliferate. Chronic antigen persistence exhausts the single target CAR T cells as well as endogenous CD8^+^ T cells and prevents differentiation of a central memory endogenous T cell pool. (**B**) BiCisCAR T cells secrete increased levels of cytokines compared with single target CAR T cells. These cytokines can promote chemotaxis of inflammatory cells from the bone marrow. BiCisCAR T cells also promote apoptosis with antigen spilling and a robust local immune response. Antigen presenting cells (APCs) activate at the tumor site and migrate to the lymph nodes to prime endogenous T cells. BiCisCAR T cells with low checkpoint expression stimulate an expanded endogenous T cell response, which persists as central memory T cells, enabling tumor control.

Tian and colleagues screened for potent CARs against neuroblastoma tumor antigens glypican-2 (GPC2 [also called CT3]) and CD276 (also called MGB7H3-LH or B7-H3) using methods to quantify and characterize surface proteins, including digital droplet PCR, pooled competitive optimization of CAR by cellular indexing of transcriptomes and epitopes–sequencing (CITE-sequencing; referred to as P-COCC in Tian et al.), and cytotoxicity assays. Having identified the most effective CARs against each of the 2 TAAs, the authors engineered BiCisCAR T cells to express CARs against both GPC2 and CD276. Since combined expression of GPC2 and CD276 is observed in 95% of neuroblastoma samples, these BiCisCAR T cells have specificity for a greater proportion of cancer cells compared to single target CAR T cells. Compared with single target CAR T cells, BiCisCAR T cells not only had comparable in vitro cytotoxicity against neuroblastoma cells coexpressing GPC2 and CD276, but they also secreted greater amounts of cytokines, such as IFN-γ and TNF-α. Thus, an advantage of BiCisCAR T cells over single target CAR T cells is their ability to initiate a robust, local inflammatory immune response, promoting recruitment and activation of other arms of the immune system, such as macrophages and dendritic cells (7) (Figure 1).

Since tumor escape due to immunoediting is a key challenge to implementing CAR T cells in solid tumors, Tian and colleagues examined the ability of BiCisCAR T cells to overcome heterogenous antigen expression by using neuroblastoma cell lines that had CRISPR mediated ablation of GPC2 or CD276. While GPC2 CARs lose cytotoxicity in the context of GPC2 ablation and CD276 lose cytotoxicity in the context of CD276 ablation, BiCisCARs retained cytotoxicity with both GPC2KO and CD276KO neuroblastoma cells. A particularly striking example of BiCisCAR T cells’ ability to prevent tumor escape was demonstrated with a heterogeneous metastatic model, where NALM6 leukemia cells expressing either GPC2 or CD276 were mixed in a 1:1 ratio and injected into mice. Both GPC2 and CD276 CAR T cells failed to control tumor growth, due to the dominance of CD276^+^ and GPC2^+^ cells respectively, whereas the BiCisCAR T cells were able to completely eradicate leukemia and prevent recurrence (7).

Effective CAR T cell therapy depends on cytotoxicity as well as long-term persistence in the body while maintaining resistance to induction of exhaustion programs. BiCisCARs were superior to single target CARs due to greater persistence in the spleen and reduced expression of PD-1, LAG-3 and TIM-3 on the CAR T cell surface 21 days after infusion. As the authors demonstrate, the higher degree of persistence may be due to a more central memory phenotype (7).

## Supercharging CAR T cells in solid tumors

The findings reported by Tian and colleagues provide important insights into how effective CAR T cell therapies can be developed for solid tumors (7). Greater persistence of BiCisCARs compared with coinjected single target CARs suggest a unique pattern of differentiation that occurs with dual CAR stimulation, which generates a more robust central memory pool and also prevents T cell exhaustion. This beneficial consequence of using dual CARs may obviate the need for checkpoint blockade or ablation, as recent findings indicate that PD-1 ablation from CAR T cells paradoxically diminishes survival and increases exhaustion — despite increased activation (9) — and may nonetheless promote accumulation of exhausted T cells (10). Chronic antigen stimulation has been shown to cause prolonged demethylation of the *PDCD1* locus, while differentiation into memory cells is associated with remethylation (11). It is likely that by clearing a tumor rapidly, dual CARs prevent the increase in PD-1 expression associated with chronic antigen stimulation, thereby preventing exhaustion of CAR T cells and allowing robust central memory differentiation. This effect could make BiCisCARs promising candidates in solid tumors that have poor response to checkpoint blockade. The enhanced cytokine secretion observed in BiCisCARs may also prove useful in increasing inflammation in the TME, thus facilitating infiltration by endogenous antitumor immune cells.

In contrast to animal models in which tumors are often eradicated quickly, limiting the opportunity for antigenic shift and tumor escape, clinical tumor regression is often a slower process. As a result, antigen-persistence could expose BiCisCAR T cells to similar exhaustion pathways observed in clinical trials using CAR T cells (12, 13). Since metabolic dysfunction precedes exhaustion of cytokine secretion and cytotoxicity (14), metabolic function could be used both as an early assessment of tolerogenic response among infused CAR T cells and to enhance CAR T cell activity. For example, Ho et al. recently showed that competing glycolysis by tumor cells limits T cell calcium-NFAT signaling by reducing the availability of glucose and its intermediate phosphoenolpyruvate (PEP) (15). Their data provided proof of concept that adoptively transferred tumor-infiltrating lymphocytes with overexpression of phosphoenolpyruvate carboxykinase 1 (PCK1) and greater levels of PEP have enhanced antitumor response and are immune from glucose-deprivation conditions. Therefore, assessing parameters such as metabolic exhaustion and engineering adaptations to the unique TME conditions of solid tumors, such as hypoxia and nutrient deprivation, may further improve the efficacy of BiCisCAR T cells against multiple tumor types.

## Version 1 08/15/2022

Electronic publication
